# Social Support Groups in the Maintenance of Glycemic Control after Community-Based Intervention

**DOI:** 10.1155/2016/7913258

**Published:** 2016-08-03

**Authors:** Claire Townsend Ing, Guangxing Zhang, Adrienne Dillard, Sheryl R. Yoshimura, Claire Hughes, Donna-Marie Palakiko, Bridget Puni Kehauoha, Ka‘imi A. Sinclair, Joseph Keawe‘aimoku Kaholokula

**Affiliations:** ^1^Department of Native Hawaiian Health, John A. Burns School of Medicine, University of Hawai‘i at Mānoa, 651 Ilalo Street, MEB 307L, Honolulu, HI 96813, USA; ^2^Office of Biostatistics & Quantitative Health Sciences, John A. Burns School of Medicine, University of Hawai‘i at Mānoa, 651 Ilalo Street, MEB 211, Honolulu, HI 96813, USA; ^3^Kula no na Po‘e Hawai‘i, P.O. Box 2368, Honolulu, HI 96823, USA; ^4^Kōkua Kalihi Valley Comprehensive Family Services, 2239 North School Street, Honolulu, HI 96819, USA; ^5^Hawai‘i Maoli, Association of Hawaiian Civic Clubs, P.O. Box 3866, Honolulu, HI 96812, USA; ^6^Ke Ola Mamo, Dillingham Plaza, 1505 Dillingham Boulevard No. 205, Honolulu, HI 96817, USA; ^7^College of Nursing, Washington State University, 1100 Olive Way, Suite 1200, Seattle, WA 98101, USA

## Abstract

Native Hawaiians and other Pacific Islanders (NH/PI; e.g., Samoan and Chuukese) have higher type 2 diabetes prevalence compared to other groups in Hawai‘i. Partners in Care (PIC), a culturally tailored, community-based, diabetes self-management education intervention (DSME), is effective at improving participants' glycemic control and self-care behaviors. Maintenance of improvements is challenging. Diabetes-related social support groups (SSG) are a promising maintenance component for DSME. This study examined the effects of a diabetes-specific SSG component relative to a control group, after the receipt of the 3-month PIC intervention, which was delivered to 47 adult NH/PI with type 2 diabetes. Participants were then randomized to either a 3-month, 6-session SSG or a control group. Hemoglobin A1c (HbA1c), blood pressure, triglycerides, cholesterol, and diabetes self-management knowledge and behaviors were assessed at baseline, 3 months, and 6 months. Results indicated significant improvements in HbA1c, diabetes-related self-management knowledge, and behaviors from baseline to 3-month assessment. However, no differences between the SSG and control group from 3-month to 6-month assessment suggest that all participants were able to maintain initial improvements. The SSG group had a significant decrease in systolic blood pressure from 3-month to 6-month assessment while the control group did not. Study limitations and future directions are discussed.

## 1. Introduction

Type 2 diabetes is a public health concern across the United States, with certain ethnic groups bearing a disproportionate burden [1, 2]. Native Hawaiians and other Pacific Islanders (NH/PI; e.g., Samoan, Chuukese) have higher type 2 diabetes incidence and prevalence compared to other ethnic groups [3, 4]. They are two times more likely to die from diabetes than the general population and suffer from high rates of diabetes-related medical complications and preventable hospitalization [5, 6]. Addressing the burden of type 2 diabetes is a priority in eliminating health disparities among NH/PI [7].

Culturally relevant, diabetes self-management interventions are important in treating type 2 diabetes among NH/PI [4, 8, 9]. Sinclair et al. found that a culturally adapted diabetes self-management intervention, called Partners in Care (PIC), significantly improved glycemic control and diabetes self-care behaviors in NH/PI compared to a wait-list control [10]. Despite the effectiveness of diabetes self-management education intervention, the maintenance of improved glycemic control continues to be a challenge across ethnic groups [11]. The long-term, postintervention maintenance of optimal glycemic control is important in judging an intervention's effectiveness [12].

Diabetes-related social support groups for those with type 2 diabetes have shown promise as a maintenance component for diabetes self-management interventions to improve long-term glycemic control and diabetes-related psychosocial functioning, self-care activities, and quality of life [13, 14]. Diabetes-related social support can include four types: appraisal support (e.g., alternative perspectives of stressors), informational support (e.g., knowledge), emotional support (e.g., expression of care), and tangible support (e.g., providing material help) [15].

The incorporation of a diabetes-related social support group for NH/PI as a maintenance component to a diabetes self-management intervention is also consistent with their shared ethnocultural values and preferences for group-based interactions [16]. They often rely on their immediate and extended family network (e.g., friends and neighbors) for emotional, physical, and spiritual support and daily decision-making [17]. Group participation with other NH/PI offers a safe and supportive environment that can increase the cultural relevance of activities and participation and enhances diabetes self-care.

To examine the effects of a diabetes-specific social support maintenance component, the community-academic partnership, the PILI ‘Ohana Project (POP), involved in Sinclair et al.'s study conducted another study of PIC with an added social support component that emphasized the four types of support [10]. The POP partnership designed a 3-month, 6-session, semistructured support group (SSG) to reinforce positive changes made during the 3-month PIC intervention. Specifically, the maintenance effects of a novel SSG on HbA1c control and diabetes self-care behaviors were examined against a control group in a sample of NH/PI with type 2 diabetes who were randomized into these conditions following their completion of PIC.

## 2. Methods

### 2.1. Participant Recruitment

**Figure 1:**
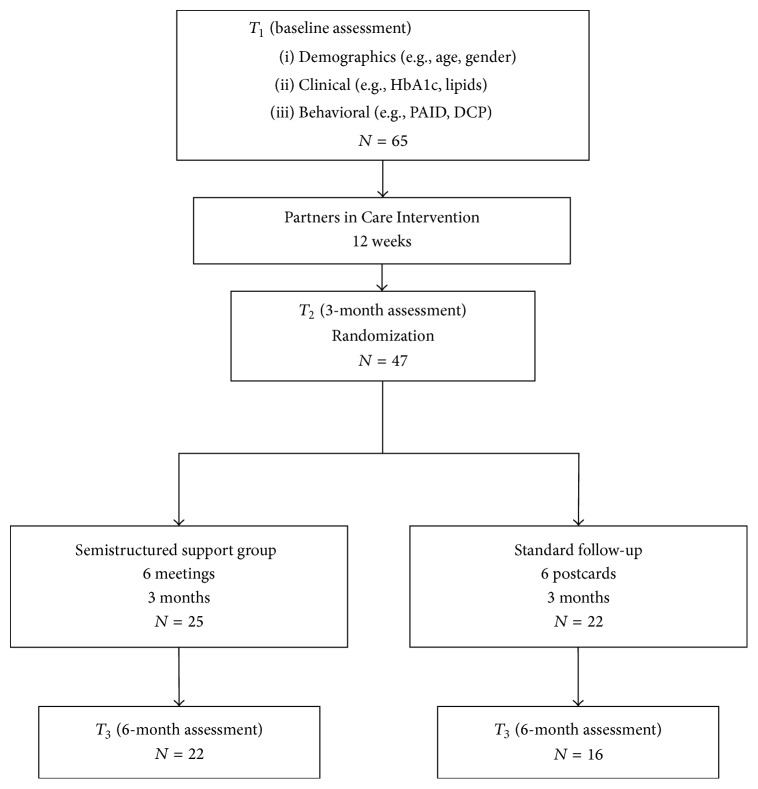
CONSORT diagram of PIC social support group study participation.

The Institutional Review Boards of the Native Hawaiian Health Care Systems and University of Hawai‘i at Mānoa approved this study. Community researchers recruited NH/PI from their respective communities and the larger NH/PI population on the Island of Oahu. Eligibility criteria were HbA1c >7%, NH/PI ethnicity, age ≥18 years, and physician-diagnosed type 2 diabetes. Eligible participants provided consent and baseline assessments (*T*_1_) were done just prior to starting PIC. The study design is shown in Figure 1.

### 2.2. Intervention and Study Procedures

PIC involves 12, 1-hour weekly group meetings, providing information on diabetes self-management and encouraging participants to work with their diabetes team that includes the individual, their family, physician, and other diabetes experts (e.g., certified diabetes educator). The intervention is based on the American Diabetes Association and the National Diabetes Education Program guidelines. PIC was culturally adapted for NH/PI based on information from focus groups with NH/PI living with diabetes and NH/PI community leaders as described in Sinclair et al. [10].

The community partners included Kula no na Po‘e Hawai‘i (a nonprofit serving urban Hawaiian Homesteads), Hawai‘i Maoli (a nonprofit serving the Hawaiian Civic Clubs), Ke Ola Mamo (the Native Hawaiian Health Care System for Oahu), and Kōkua Kalihi Valley (a health clinic serving low-income PI). These community organizations are described in detail by Nacapoy et al. [18]. The community partners recruited participants, delivered the intervention, and conducted the baseline and outcomes assessments at their respective organizations. All participants completed a baseline assessment (*T*_1_), received PIC, and underwent a second assessment at 3 months (*T*_2_). The protocol used at each assessment and measures were the same as used by Sinclair et al. [10]. Following assessment at *T*_2_, participants were randomized, based on a 1 : 1 randomization by site, to either the 3-month SSG or standard follow-up control group.

Participants randomized to the SSG attended six bimonthly, semistructured group meetings, lasting for about 1 hour, to reinforce skills taught in PIC. Trained community facilitators (CF) led two of the sessions and health professionals (i.e., pharmacist, nutritionist, physician, and psychologist) led the remaining four sessions. Community facilitators were instructed to provide appraisal and emotional support (e.g., talking through difficulties and encouraging connection between group members) on how to garner additional support from family/friends for diabetes self-management activities (i.e., healthy eating, physical activity, and medication adherence). The health professionals concentrated on providing informational and appraisal support around managing diet, medications, diabetes-related complications, and maintaining self-care activities. The control group received only six bimonthly postcards reminding them of performing diabetes self-management activities. All participants underwent a final assessment at *T*_3_ after the 3-month maintenance component (i.e., six months after *T*_1_).

### 2.3. Measures

#### 2.3.1. Primary Outcome Measures

Clinical measures included HbA1c, measured with the Bayer DCA 2000 via a fingerstick sample of whole blood. The same blood sample was used to measure total cholesterol, high-density lipoprotein (HDL), and low-density lipoprotein (LDL) and triglycerides with the Cholestech LDX lipid profile system. Blood pressure, weight (kg), and height (cm) were measured twice at each assessment with the average of the two values used in the analysis.

#### 2.3.2. Secondary Outcome Measures

The understanding subscale of the diabetes care profile (DCP) measured understanding of diabetes self-care activities [19]. It consists of 12 items with a 1 (poor understanding) to 5 (excellent understanding) Likert-type response scale. The scores for the 12 items were averaged to yield a total score between 1 and 5. Higher scores indicate greater understanding. Seven of the 11 items from the Summary of Diabetes Self-Care Activities (SDSCA) were used to measure the frequency with which participants conducted self-care activities (e.g., checked their feet) during the previous week [20]. The scoring for each item was as follows: 1 (not at all during the past 7 days), 2 (2-3 days), 3 (4–6 days), and 4 (7 days). The summed total scores ranged from 7 to 28. Higher scores indicate greater frequency of self-care activities. The 20-item problem areas in diabetes (PAID) assessed quality of life such as physical/social functioning and mental/emotional well-being specific to living with diabetes [21]. The possible responses to each item ranged from 0 (not a problem) to 4 (serious problem). The total score was the sum of all items multiplied by 1.25 so that scores ranged from 0 to 100. Higher scores indicate greater diabetes-related emotional distress.

### 2.4. Statistical Analysis

Demographic and clinical measures were summarized by frequencies and percentages for categorical variables and means (M) and standard deviations (SD) for continuous variables. Independent two sample *t*-tests were used to examine changes within subject. Support and control groups were compared using Chi-square or Fisher's exact test when appropriate for continuous and categorical variables. Analysis of covariance (ANCOVA) was used to test between group differences at *T*_2_ and *T*_3_, adjusting for between-group differences at *T*_1_ and *T*_2_, respectively. Statistical analyses were performed using SAS software version 9.4 (SAS Institute Inc., Cary, NC, USA). A *p* value < 0.05 is statistically significant.

## 3. Results

### 3.1. Baseline and *T*_2_ Characteristics

The baseline characteristics for the 47 NH/PI receiving the PIC intervention are summarized in Table 1. It indicates that, among the participants, slightly over half were female, married, and Native Hawaiian and had a high school diploma or its equivalent. Participants on average had BMI in the severely obese category (M = 36.01 ± 6.77), blood pressure in the prehypertensive range (SBP M = 129.59 mmHg ± 15.77; DBP M = 76.46 mmHg ± 11.00), and mean HbA1c of 9.98 ± 2.23. Although mean total cholesterol (M = 183.45 mg/dL ± 43.77) and LDL cholesterol (M = 93.36 mg/dL ± 38.49) were within the recommended range, participants had low HDL cholesterol (M = 40.72 mg/dL ± 13.40) and high triglyceride levels (M = 240.59 ± 171.07).

Table 1 also summarizes participant characteristics by group at 3-month assessment (*T*_2_). At *T*_2_, both the SSG and control group had mean BMIs that remained in the severely obese category (M = 37.27 ± 7.66 and M = 35.42 ± 4.63, resp.). The SSG had slightly higher mean systolic (M = 137.48 ± 24.81) and diastolic blood pressure (M = 81.72 ± 14.22) but lower HbA1c (M = 8.96 ± 1.82) compared to the control group (M = 132.03 ± 21.43, M = 76.50 ± 12.96, M = 9.47 ± 2.69, resp.). However, none of these differences between groups at *T*_1_ or *T*_2_ were statistically significant.

### 3.2. Pre- and Post-PIC Intervention Outcomes

#### 3.2.1. Combined Sample

Data in Table 2 shows the mean changes in behavioral and biological measures across three assessment periods and for the combined sample for both the complete case and the intent-to-treat analysis. In the complete case analysis, there were significant improvements in the following variables from *T*_1_ to *T*_2_: HbA1c (M = −0.76 ± 1.86, *p* < 0.01), DCP (M = 0.73 ± 0.97, *p* < 0.001), PAID (M = −11.1 ± 21.87, *p* < 0.001), and SDSCA (M = 2 ± 5.12, *p* < 0.01). Except for HbA1c, significant improvements in these variables were also found from *T*_1_ to *T*_3_. Examining change between *T*_2_ and *T*_3_ shows a significant increase in LDL (M = 13.55 mg/dL ± 26.42, *p* < 0.05), decrease in SBP (M = −7.62 mmHg ± 16.6, *p* < 0.05), and increase in SDSCA (M = 1.7 ± 4.67, *p* < 0.05). The intent-to-treat analysis provided similar results, with the exception of change in HbA1c from *T*_1_ to *T*_3_, which showed a significant decrease (M = −0.53 ± 1.80, *p* < 0.05).

#### 3.2.2. Social Support Group versus Control

A comparison of the mean changes in variables between *T*_2_ and *T*_3_ by group is presented in Table 3. At *T*_2_, 25 participants were randomized to the SSG and 22 to the control group, with 22 and 12 participants being retained at *T*_3_, respectively. There were no significant differences in the changes in variables between the SSG and control group from *T*_2_ to *T*_3_, controlling for *T*_2_ values. There was a statistically significant reduction in SBP in the SSG (M = −8.36 mmHg ± 16.22, *p* = 0.025) but not in the control group (M = −6.25 mmHg ± 17.93, *p* = 0.253). There were marginally significant improvements in DCP (M = −0.24 ± 0.55, *p* = 0.054) and SDSCA (M = 1.41 ± 3.49, *p* = 0.072) scores in the SSG but not in the control group (M = −0.12 ± 0.80, *p* = 0.621, and M = 2.27 ± 6.62, *p* = 0.281, resp.).

## 4. Discussion

Type 2 diabetes is a serious threat to the health and well-being of NH/PI as culturally tailored, diabetes self-management interventions, such as PIC, can help attenuate. The 12-week PIC intervention led to significant improvements in HbA1c, diabetes self-care knowledge and activities, and emotional well-being. However, we did not find significant differences in the maintenance of these improvements between participants randomized to either the SSG or control group following completion of PIC. Participants' glycemic control at 6 months was not significantly different from their control immediately after PIC. This suggests that participants were able to maintain initial improvements from PIC with or without the SSG. While not significantly different between groups, the SSG group had a significant within-group decrease in systolic blood pressure from *T*_2_ to *T*_3_ while the control group did not. The SSG also had improvements in understanding of diabetes and frequency of self-care activities that were marginally significant.

Although this study did not support the hypothesis that SSG can improve the maintenance of glycemic control after intervention, we did find some improvements in other outcomes (e.g., systolic blood pressure). To date, the literature on social support and HbA1c is mixed. The findings of our research suggest that social support alone may not reduce HbA1c. Our results are consistent with other studies that found modest improvements in diabetes understanding and self-care activities but no change in HbA1c [14, 22].

Our results indicate that the social support provided to the SSG may have helped to improve their systolic blood pressure. A similar study in African Americans found that despite no improvements in HbA1c after a 3-month diabetes self-management intervention, participants randomized to a 12-month social support group had significant improvements in systolic blood pressure while the control group did not [23]. This finding is important given that over time cardiovascular disease risk factors, such as systolic blood pressure, tend to worsen [24]. Additionally, the UKPDS study found that maintaining blood pressure in the normal range resulted in an 11% decrease in diabetes complications over 10 years [25]. Other studies have found that intensive blood pressure control can save approximately $2,000 per quality-adjusted life-year in patients with type 2 diabetes [26].

Despite mixed findings in the research on the impact of social support on HbA1c in patients with diabetes, the association between social support and blood pressure is well established [27]. Based on communication with community researchers, there is a belief that social support groups can help to build relationships among community members and encourage interaction outside of the intervention. This could provide participants with a sense of accountability and opportunities to learn from each other, which may increase motivation to maintain positive behavior changes and improve psychosocial functioning [14, 28]. Thus, the use of social support groups remains a preference in our communities.

Our study has several limitations relevant to the SSG component. The sample size may have been too small to detect between group differences. Also, participants in the control group received bimonthly postcards reminding them of the skills they learned in the PIC intervention. These postcards may have been effective at helping participants maintain the self-care activities they initiated during the intervention, lessening any between group differences at *T*_3_. As a RCT, participants were randomized after the 12-week PIC intervention. Due to the fact that several of these groups were small (e.g., 8 people), the number of people randomized to SSGs was very small, which may have limited the amount of support each group was able to provide. Additionally, some participants formed relationships in PIC but were separated by randomization into different groups, which possibly decreased the motivation of these participants. The structure of the SSG was set a priori; however some participants expressed an interest in diabetes-related topics not included and/or in an order different from what was scheduled, which may have caused participants to lose interest.

Our study concurs with the review done by Tomioka et al., in which they state that future research on the use of social support groups in improving HbA1c and blood pressure is necessary, a belief with which the community agrees [9]. The use of RCTs in which participants are randomized at the individual level after intervention may not be an appropriate design in testing support group components. Future designs could randomize by community site, allowing relationships built during the intervention to continue during support groups. Other recommendations include the use of support groups that occur on an ongoing basis facilitated by health professionals with diabetes expertise. Consequently, participants could attend as they feel necessary and exercise control in determining topics discussed. In conclusion, the PIC diabetes self-management intervention is effective at decreasing participants' HbA1c and improving their self-management skills. However, maintaining improvements in HbA1c warrants further research.

## Figures and Tables

**Table 1:** Participants' sociodemographic, behavioral, and biological characteristics for combined sample at *T*_1_ and by group at *T*_2_.

Variable	Baseline = *T*_1_	3 months = *T*_2_	
	Total (*N* = 47)	SSG (*N* = 25)	Control (*N* = 22)
Age (years)	54.53 ± 10.18	54.62 ± 11.06	54.42 ± 9.29
Sex			
Female	23 (50)	10 (40)	13 (62)
Ethnicity			
Hawaiian	27 (57)	14 (56)	13 (59)
Micronesian	16 (34)	8 (32)	8 (36)
Filipino	2 (4)	2 (8)	0 (0)
Other	2 (4)	1 (4)	1 (5)
Education			
Less than high school	6 (13)	2 (8)	4 (19)
High school diploma/GED	27 (60)	16 (67)	11 (52)
Some college/tech	10 (22)	5 (21)	5 (24)
College degree	2 (4)	1 (4)	1 (5)
Marital status			
Never	5 (11)	4 (16)	1 (5)
Currently	26 (58)	14 (56)	12 (60)
Disrupted	14 (31)	7 (28)	7 (35)
Weight (kg)	100.77 ± 24.39	106.42 ± 28.36	97.05 ± 12.90
BMI (kg/m^2^)	36.01 ± 6.77	37.27 ± 7.66	35.42 ± 4.63
HbA1c (%)	9.98 ± 2.23	8.96 ± 1.82	9.47 ± 2.69
Cholesterol (mg/dL)	183.45 ± 43.77	171.79 ± 36.82	171.24 ± 38.80
LDL cholesterol (mg/dL)	93.36 ± 38.49	92.38 ± 37.84	81.36 ± 37.41
HDL cholesterol (mg/dL)	40.72 ± 13.40	42.00 ± 14.90	38.33 ± 7.34
Triglycerides (mg/dL)	240.59 ± 171.07	234.00 ± 175.38	268.19 ± 142.08
Systolic blood pressure (mmHg)	129.41 ± 15.77	137.48 ± 24.81	132.03 ± 21.43
Diastolic blood pressure (mmHg)	76.46 ± 11.00	81.72 ± 14.22	76.50 ± 12.96
Diabetes care profile score	2.93 ± 0.86	3.55 ± 0.80	3.52 ± 0.87
Problem areas in diabetes score	34.41 ± 23.43	26.80 ± 20.70	26.46 ± 27.52
Summary of diabetes self-care activities score	17.00 ± 4.81	18.52 ± 4.12	18.06 ± 5.02

Note. Body mass index is abbreviated as BMI, high-density lipoprotein as HDL, low-density lipoprotein as LDL, and social support group as SSG. HbA1c is the measure of glycated hemoglobin.

Data shown as mean ± SD or *n* (%).

No significant differences between SSG and control group at *T*_1_ or *T*_2_, all *p* values > 0.15.

**Table 2:** Mean change in behavioral and biological measures across three assessments for the combined sample.

Variable	*T*_1_ to *T*_2_ (*N* = 47)	*T*_2_ to *T*_3_ (*N* = 34)	*T*_1_ to *T*_3_ (*N* = 38)
Weight (kg)	0.08 ± 4.97	5.41 ± 22.13	4.96 ± 21.36
ITT weight (kg)	0.44 ± 5.11	0.27 ± 2.13	0.71 ± 5.49
BMI (kg/m^2^)	0.08 ± 1.78	2.09 ± 8.16	2 ± 7.82
ITT BMI (kg/m^2^)	0.22 ± 1.82	0.11 ± 0.75	0.33 ± 1.91
HbA1c (%)	−0.76 ± 1.86^*∗∗*^	0.24 ± 1.14	−0.57 ± 1.88
ITT HbA1c (%)	−0.73 ± 1.80^*∗∗*^	0.17 ± 1.02	−0.53 ± 1.80^*∗*^
Cholesterol (mg/dL)	−10.7 ± 37.73	4.4 ± 27.92	−1.74 ± 52.59
ITT cholesterol (mg/dL)	−11.38 ± 36^*∗*^	3.14 ± 23.57	−5.43 ± 49.94
LDL cholesterol (mg/dL)	−6.25 ± 31.5	13.55 ± 26.42^*∗*^	6.73 ± 36
ITT LDL cholesterol (mg/dL)	−5.94 ± 29.05	7.32 ± 20.38^*∗*^	5.82 ± 35.09
HDL cholesterol (mg/dL)	1.39 ± 15.15	−0.67 ± 8.46	−0.77 ± 11.93
ITT HDL cholesterol (mg/dL)	−0.22 ± 11.24	−0.45 ± 6.91	−15.20 ± 168.24
Triglycerides (mg/dL)	−1.24 ± 170.99	−30.83 ± 160.55	−37.87 ± 170.51
ITT triglycerides (mg/dL)	9.68 ± 151.76	−21.8 ± 135.08	−15.20 ± 168.24
Systolic blood pressure (mmHg)	2.59 ± 20.43	−7.62 ± 16.6^*∗*^	−2.28 ± 16.07
ITT systolic blood pressure (mmHg)	4.95 ± 19.47	−6.02 ± 15.05^*∗*^	0.00 ± 17.25
Diastolic blood pressure (mmHg)	2.61 ± 12.05	−3.34 ± 12.46	0.61 ± 11.62
ITT diastolic blood pressure (mmHg)	3.16 ± 11.48	−2.64 ± 11.13	0.65 ± 11.44
Diabetes care profile	0.73 ± 0.97^*∗∗∗*^	−0.2 ± 0.64	0.39 ± 0.99^*∗*^
ITT diabetes care profile	0.65 ± 1.00^*∗∗∗*^	−0.16 ± 0.57	0.48 ± 1.04^*∗∗*^
Problem areas in diabetes	−11.1 ± 21.87^*∗∗∗*^	1.51 ± 11.53	−7.04 ± 18.21^*∗*^
ITT problem areas in diabetes	−8.64 ± 20.2^*∗∗*^	1.19 ± 10.24	−7.93 ± 18.63^*∗∗*^
Summary of diabetes self-care activities	2 ± 5.12^*∗∗*^	1.7 ± 4.67^*∗*^	2.94 ± 5.54^*∗∗*^
ITT summary of diabetes self-care activities	1.59 ± 5.11^*∗*^	1.27 ± 4.10^*∗*^	2.74 ± 5.26^*∗∗*^

Note: Data shown as mean ± SD. Baseline = *T*_1_, 3-month assessment = *T*_2_, and 6-month assessment = *T*_3_. Body mass index is abbreviated as BMI, high-density lipoprotein as HDL, and low-density lipoprotein as LDL. A1c is the measure of glycated hemoglobin. Values are expressed as mean ± SD. Significance in change within group during the specified time period is tested by paired *t*-test and denoted by ^*∗*^ *p* < 0.05, ^*∗∗*^ *p* < 0.01, and ^*∗∗∗*^ *p* < 0.001.

**Table 3:** Mean change in behavioral and biological measures from *T*_2_ to *T*_3_ by group.

Variable	SSG (*N* = 22)	Control (*N* = 12)
Weight (kg)	0.19 ± 2.28	0.64 ± 2.78
BMI (kg/m^2^)	0.11 ± 0.76	0.20 ± 1.07
HbA1c (%)	0.35 ± 1.11	−0.04 ± 1.12
Cholesterol (mg/dL)	5.33 ± 26.62	3.00 ± 30.93
LDL cholesterol (mg/dL)	12.75 ± 29.80	14.75 ± 22.30
HDL cholesterol (mg/dL)	0.47 ± 6.66	−2.60 ± 11.01
Triglycerides (mg/dL)	−17.72 ± 174.9	−52.27 ± 139.14
Systolic blood pressure (mmHg)	−8.36 ± 16.22^*∗∗*^	−6.25 ± 17.93
Diastolic blood pressure (mmHg)	−3.02 ± 11.45	−3.92 ± 14.65
Diabetes care profile score	−0.24 ± 0.55^*∗*^	−0.12 ± 0.80
Problem areas in diabetes score	2.50 ± 9.71	−0.31 ± 14.61
Summary of diabetes self-care activities	1.41 ± 3.49^*∗*^	2.27 ± 6.62

Data shown as mean change ± SD. Significance in change is tested by paired *t*-test and denoted by ^*∗*^ *p* < 0.1 and ^*∗∗*^ *p* < 0.05.
